# Supplementary material for: Ozonated Sunflower Oil Embedded within Spray-Dried Chitosan Microspheres Cross-Linked with Azelaic Acid as a Multicomponent Solid Form for Broad-Spectrum and Long-Lasting Antimicrobial Activity
Source: Pharmaceutics. 2024 Apr 6;16(4):502. doi: 10.3390/pharmaceutics16040502 (PMC11054446; doi:10.3390/pharmaceutics16040502)
Supplement: Supplementary file 1 [file pharmaceutics-16-00502-s001.zip › pharmaceutics-2920365-supplementary.pdf]

Article

Supplementary Materials: Ozonated Sunflower Oil Embedded within Spray-Dried Chitosan Microspheres Cross-Linked with Azelaic Acid as a Multicomponent Solid Form for Broad-Spectrum and Long-Lasting Antimicrobial Activity

Roberto Spogli, Caterina Faffa, Valeria Ambrogi, Vincenzo D'Alessandro and Gabriele Pastori

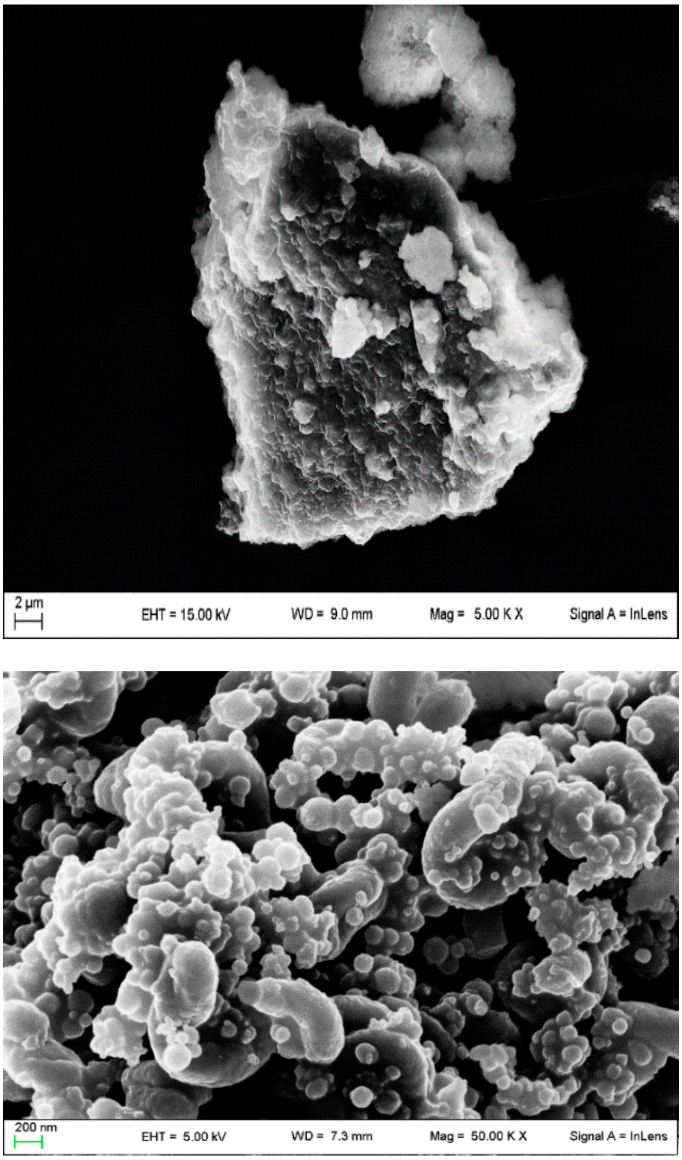

Figure S1. SEM pictures of samples Si@OZ,.

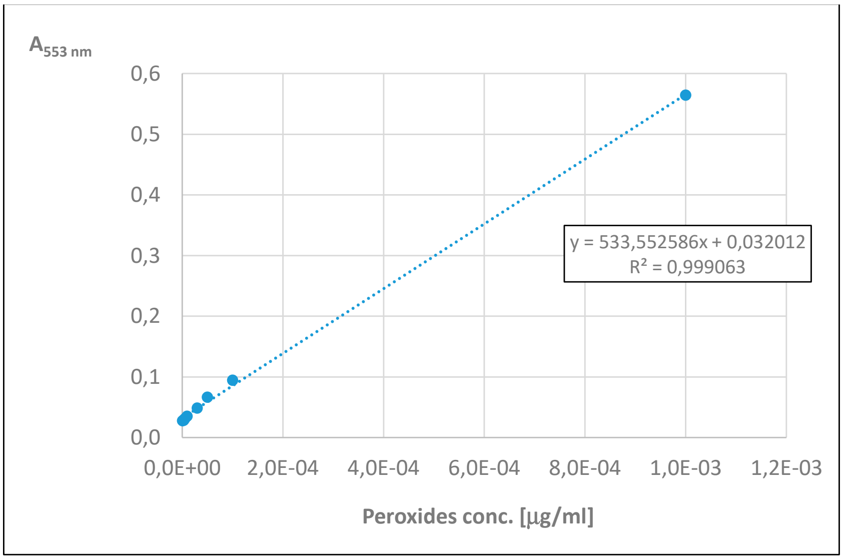

Figure S2. plot of H_2O_2 in water and in 0.5 M pH 4 acetate buffer.

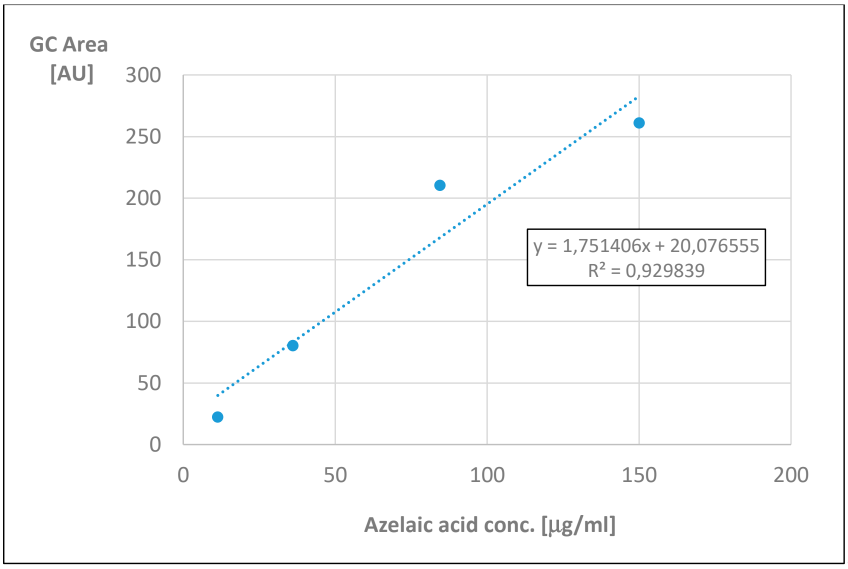

Figure S3. calibration plot of azelaic acid treated with diazomethane.

Table S1. measurement of mmol peroxides over time.

	10^{-3} moles of peroxides equivalent					
**Time h**	**OZ**	**Si@OZ**	**CH-MS-OZ**	**CH-MS-Si@OZ**	**CH-MS**	**Si@**
1	1.90	2.63	4.02	0.75	0.67	0.22
4	2.27	2.98	3.51	0.60	0.33	0.68
7	2.26	2.78	2.64	0.90	0.25	0.68
24	5.09	3.48	4.90	3.45	0.21	0.69
48	8.44	3.78	7.13	3.49	0.65	0.65
96	17.30	4.54	6.45	2.94	0.68	0.69
144	31.50	5.15	10.40	5.84	0.54	0.68
168	34.50	5.53	12.30	6.12	0.21	0.54

The error on each measurement is 8% as determined by the standard error associated to the linear regression analysis.

Table S2. GC analysis of four standard solutions of azelaic acid.

	**Azelaic acid concentration (stoichiometric)**	**Azelaic acid concentration (found)**	**Deviation**
	μg/ml	μg/ml	[%]
STANDARD A	**150**	123.7838	−17.5
STANDARD B	**10**	9.3281	−6.7
STANDARD C	**75**	69.6797	−7.1
STANDARD D	**30**	29.7545	−0.8
AVERAGE			8.0 %

Method S1: determination of peroxides through DPD oxidation UV-VIS spectrophotometric method

A calibration plot of H_2O_2 in water and in 0.5 M pH 4 acetate buffer (release medium) was firstly obtained by analysing H_2O_2 standards at various known concentrations (in the range 10^{-6} M - 10^{-3} M), then samples obtained from the release test were analysed.

Briefly, 50 μl samples/standard were added after vigorous vortexing with 1.8 ml of 0.5 M pH 3 acetate buffer, 150 μl of 25 mM iron sulphate solution and 0.5 ml of 100 mM DPD solution in a 1 cm length vial. The mixture was stirred for 25 seconds and at time 45 seconds analysed through UV-vis spectrometer. The spectra were corrected for the baseline between 800 and 700 nm and Absorbance value corresponding to 553 nm (A_{553}) was determined.

A UV-vis spectrum of the mixture without sample (sample replaced by 50 μl of ultrapure water) was always recorded as background. Samples without OZ (Si@ and CH-MS) were analysed too, as negative control whereas samples containing different concentration of pure OZ were used as positive control.

0.5 M pH 3 and pH 4 acetate buffer was prepared by AcOH and sodium acetate, 25 mM iron sulphate solution and 100 mM DPD sulphate solution were prepared at the moment. Hydrogen peroxide standard solutions were prepared using H_2O_2 (35%w/w).

Method S2: determination of azelaic acid via derivatization and GC-analysis with internal standard

Gas-chromatography parameters: Model: Agilent 6820, Column: BD-35MS 30 m / 0.32 mm ID 0.25 μm thickness, Carrier gas: He, 1.54 ml/min 40 °C, Injector: splitless mode, 280 °C, Detector: FID, 340 °C, Temperature program: hold 3 min 40 °C, 5 °C/min up to 320°C, hold 5 mi 320 °C; Internal standard: 1-dodecanol (conc. 109.48 μg/mL)

Derivatization procedure: Azelaic acid samples have been methylated trough the reaction with diazomethane. To 0.50 ml of the azelaic acid solution to be tested and 0.10 ml of internal standard stock solution (1094.8 μg/mL), a diazomethane solution in diethyl ether (2M) have been added dropwise until a persistent yellow color is observed (about 15 drops). The solution is then brought to a final volume of 1.0 ml with 96% ethanol.

The Relative Response Factor (RRF) for the internal standard have been determined:

RRF = (AIS*CAZ)/(AAZ*CIS) = 1.29923

AIS = Gas-chromatographic area of Internal Standard

CAZ = Concentration of azelaic acid (dimethyl azelate derivatization)

AAZ = Gas-chromatographic area of azelaic acid (dimethyl azelate derivatization)

CIS = Concentration of Internal Standard

The method described above has been validated through the analysis of four standard solutions of azelaic acid.
